# Assessment of Fish Community Structure and Invasion Risk in Xinglin Bay, China

**DOI:** 10.3390/biology14080988

**Published:** 2025-08-04

**Authors:** Shilong Feng, Xu Wang, Liangmin Huang, Jiaqiao Wang, Lin Lin, Jun Li, Guangjie Dai, Qianwen Cai, Haoqi Xu, Yapeng Hui, Fenfen Ji

**Affiliations:** 1 Fisheries College, Jimei University, Xiamen 361021, China; 202312951064@jmu.edu.cn (S.F.); 202412951039@jmu.edu.cn (X.W.); lmhuang@jmu.edu.cn (L.H.); skyofstar1@jmu.edu.cn (J.W.); 202411710015@jmu.edu.cn (L.L.); lijun1982@jmu.edu.cn (J.L.); xhqcurious@163.com (H.X.); 202412951041@jmu.edu.cn (Y.H.); 2 Fujian Provincial Key Laboratory of Marine Fishery Resources and Eco-Environment, Xiamen 361021, China; 3 Agriculture, Rural and Water Resources Bureau of Jimei District, Xiamen 361022, China; daiguangjie@jimei.gov.cn (G.D.); caiqianwen@jimei.gov.cn (Q.C.)

**Keywords:** non-native fish species, environmental DNA metabarcoding, Aquatic Species Invasiveness Screening Kit, Xinglin Bay

## Abstract

A total of 32 fish species were detected in Xinglin Bay using a combination of environmental DNA metabarcoding (eDNA) and traditional morphological survey methods (TSM), covering eight orders, fifteen families, and twenty-six genera. The dominant order was Perciformes, accounting for 43.75% of the total species. Among the identified species, there were ten non-native fish species. Compared with the TSM, the eDNA detected 13 additional fish species, including two additional non-native fish species—*Gambusia affinis* (Baird and Girard, 1853) and *Micropterus salmoides* (Lacepède, 1802). In addition, the relative abundance of fish from both methods revealed that tilapia was overwhelmingly dominant, accounting for 80.75% and 75.68%, respectively. Furthermore, the AS-ISK assessment revealed that all non-native fish species were classified as medium or high-risk, with five identified as high-risk species, four of which belong to tilapia. These findings demonstrated that tilapia are the dominant and high-risk invasive species in Xinglin Bay and should be prioritized for management. Population reduction through targeted harvesting of tilapia is recommended as the primary control strategy. Additionally, the study highlights the effectiveness of eDNA in monitoring fish community structure in brackish ecosystems.

## Simple Summary

Urban waterbodies with important ecological functions are increasingly threatened by non-native fish species, particularly in densely populated and ecologically sensitive coastal areas. Xinglin Bay in Xiamen, China, is a lentic brackish ecosystem with significant ecological and scenic value that has long been threatened by non-native fish species. However, a systematic assessment of its invasion status and associated risks has yet to be conducted. This study combined the traditional morphological survey method (TSM) with environmental DNA metabarcoding (eDNA) to evaluate the structure of the fish community and the status of non-native fish species in Xinglin Bay in August 2024. Furthermore, the invasion risks associated with the identified non-native species were assessed using the Aquatic Species Invasiveness Screening Kit (AS-ISK). Our findings will aid in the prevention and management of non-native fish species in Xinglin Bay and advance the use of eDNA technology for monitoring fish communities in lentic brackish ecosystems.

## 1. Introduction

Urban waterbodies, as vital ecological infrastructure, not only serve functions such as water supply, climate regulation, and pollutant degradation but also play a crucial role in supporting urban biodiversity [1]. Compared with natural waterbodies, the success rate of non-native fish in urban waters is three to five times higher [2]. With the rapid acceleration of urbanization, approximately 65% of freshwater ecosystems worldwide are facing serious challenges from non-native fish species [3,4]. Santana et al. (2020) found that urban waterbodies in Rio de Janeiro, Brazil, had long been invaded by Poecilia reticulata, with the population density of P. reticulata in urban waters 26 times higher than that in adjacent natural habitats, and individuals being significantly larger in size [5]. Zhang et al. (2022) evaluated the fish communities and invasion status across 109 urban and rural sites in Beijing, China, identifying 52 native and 23 non-native fish species [6]. Their research found that non-native species in urban waterbodies, such as *Rhinogobius brunneus* (Temminck and Schlegel, 1845) and tilapia, exhibited greater tolerance to anthropogenic stress and tended to outcompete native species to become dominant [6]. Moreover, in major metropolitan waterbodies, such as the Seine River in Paris, the Arno River in Florence, the Maozhou, Guanlan, and Shenzhen Rivers in China, the Saugus River in the United States, and the Gravalai in Brazil, introduced alien fish species have already displaced native species and become dominant within local aquatic ecosystems [7,8,9,10]. Therefore, urban waterbodies are generally under threat from alien species, and assessing their distribution and invasion risks is fundamental for effective prevention and management of non-native fish.

Historically, the assessment of non-native fish species has predominantly employed the traditional morphological survey method (TSM) [11,12]. Despite its widespread application, this approach is often labor-intensive, presents difficulties in accurate species identification, and may overlook species that are low in abundance or highly cryptic [13]. Environmental DNA metabarcoding (eDNA) enables rapid and accurate non-native fish assessment by analyzing species-specific DNA from environmental samples, revolutionizing traditional morphology-based identification [14,15]. Although environmental DNA (eDNA) has been extensively applied in biodiversity research, the majority of existing studies have concentrated on evaluating fish community or non-native fish within freshwater and marine ecosystems [16,17,18]. In contrast, investigations utilizing eDNA to assess fish communities and the status of non-native fish species in brackish environments remain limited, particularly within lentic waterbodies where such applications are still relatively underexplored.

Additionally, as non-native fish species pose increasingly serious threats to freshwater ecosystems, corresponding risk assessment methods have continuously evolved—from early qualitative analyses based on expert judgment to quantitative models that incorporate ecological traits, environmental adaptability, and spatial distribution data [19,20]. The ecological risk prediction framework proposed by Kolar and Lodge (2002) was the first to integrate biological attributes with environmental factors, successfully predicting potential non-native species in the North American Great Lakes region [21]. In recent years, the Aquatic Species Invasiveness Screening Kit (AS-ISK) has gained widespread application in regions such as Europe and Asia for the rapid risk screening of non-native fish species, due to its ease of use and clearly defined quantitative criteria [22].

Xiamen, a key city in China’s southeast coastal special economic zone, serves as an important transportation hub, port, and tourist destination. Xinglin Bay, situated within the urban area, serves as a crucial ecological, landscape, and hydrological resource [23]. In January 2023, our team conducted the inaugural integrated assessment of the fish community structure in Xinglin Bay by combining environmental DNA barcoding and traditional survey methods, providing a preliminary characterization of the fish assemblage [24]. However, current research on the fish communities in Xinglin Bay remains limited, particularly lacking a systematic risk assessment of non-native fish species. Therefore, this study aims to (1) systematically investigate the fish community structure in Xinglin Bay using TSM coupled with eDNA, with a focus on identifying the non-native fish species and abundance, and (2) assess the invasion risks of non-native fish species using the AS-ISK. The findings of this study will provide a scientific basis for the prevention and control of non-native fish and the conservation of aquatic biodiversity in Xinglin Bay, while also offering methodological references for managing non-native fish species in other urban waterbodies.

## 2. Materials and Methods

### 2.1. Study Area

**Figure 1:**
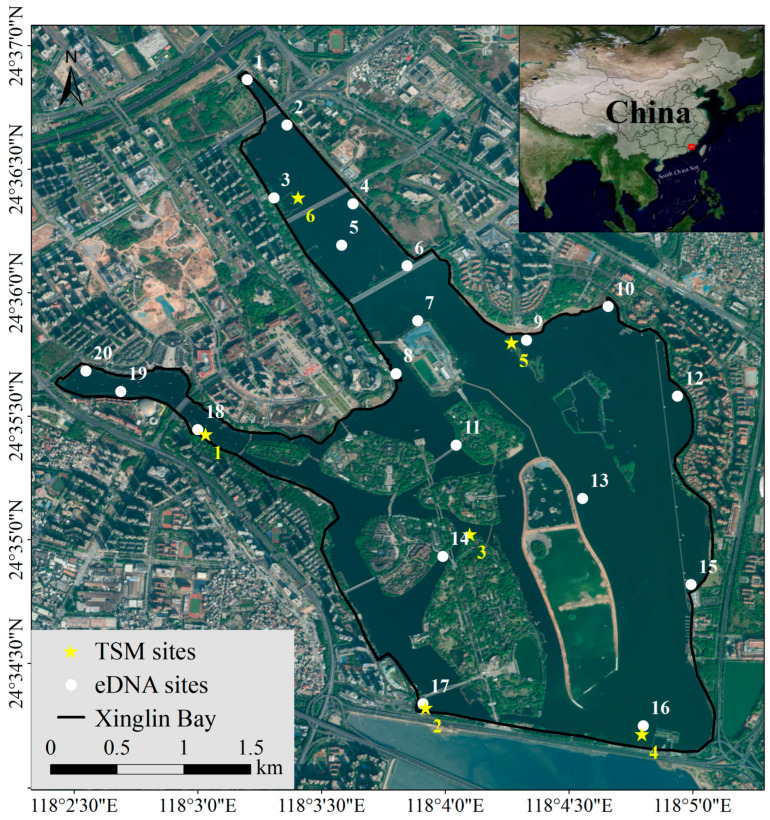
Schematic diagram of Xinglin Bay and the sampling sites.

Situated in Xiamen City, Fujian Province, China, Xinglin Bay is geographically positioned between 29°46′71″ and 29°51′45″ N latitude, and 112°31′36″ to 112°37′30″ E longitude. Xinglin Bay, encompassing an area of approximately 6 km^2^ with an average depth of 2.5 m, was formed in 1956 following the construction of the Jixing Dam [23]. The salinity progressively rises from the freshwater inflow in the north (proximal to site 1) toward the southern estuarine region (site 4 for TSM; site 16 for eDNA) of Xinglin Bay. Situated at the mouth of the Houxi River, the dam marks a key geographical point. During the August 2024 fishery resource assessment in Xinglin Bay, the TSM approach was employed at six locations, while environmental DNA sampling was conducted at twenty different sites (each eDNA sampling site will conduct three parallel experiments to ensure the accuracy of the results) (Figure 1).

### 2.2. Specimen Collection Using Conventional Survey Techniques

Fish sampling followed standard fishery resource assessment protocols [25]. Specimens were collected using gillnets and cylindrical traps [26]. At each site, two gillnets per mesh size (50.0 m × 1.5 m; mesh sizes: 28.0, 60.0, and 100.0 mm) and four traps (50.0 m × 30 cm) were deployed. The gillnets were arranged perpendicular to the shoreline and operated for three hours, while the traps operated for 24 h. Fish specimens were kept on ice and transported to the laboratory within two hours for species identification and further analysis [12]. Species identification was conducted immediately using *Fishes of Fujian* and FishBase as taxonomic references [27].

### 2.3. Environmental DNA (eDNA) Sampling and Analytical Procedures

At each site, 1 L of surface water (5–10 cm depth) was collected using a dedicated sampler and immediately stored in pre-sterilized plastic bottles. Samples were kept chilled on ice during transport and filtered within four hours post-collection [28,29]. Filtration employed sterile 0.45 μm mixed cellulose ester membranes (Millipore; JINTENG, Tianjin, China) using a vacuum filtration apparatus (YUZE, Tianjin). To prevent contamination, filtering equipment and tweezers were subjected to sodium hypochlorite treatment and thoroughly washed with molecular-grade ddH_2_O between sites. Field staff operated under strict aseptic protocols, including glove use during all procedures [29].

Following filtration, each membrane was sealed in a sterile 5 mL centrifuge tube and frozen at −20 °C until DNA extraction [29]. Genomic DNA was isolated using the MP FastDNA^®^ Spin Kit (MP Biomedicals, Santa Ana, CA, USA), with all extracts stored at −20 °C for subsequent molecular analysis. Fish taxa were identified via metabarcoding targeting a ~350 bp fragment of the mitochondrial 16S rRNA gene using Actinopterygii-specific primers (Ac16s-F: 5′-CCTTTTGCATCATGATTTAGC-3′; Ac16s-R: 5′-CAGGTGGCTGCTTTTAGGC-3′) [30].

Amplifications via PCR were carried out in 20 μL reaction mixtures, which included 4 μL of 5× FastPfu buffer, 2 μL of 2.5 mM dNTPs, 0.8 μL of each primer (5 μM), 0.4 μL of FastPfu polymerase, ~10 ng of template DNA, and nuclease-free water. Thermal cycling included the following steps: initial denaturation at 94 °C (3 min); 45 cycles of 94 °C (30 s), 60 °C (30 s), and 72 °C (30 s); and a final extension at 72 °C (5 min). PCR products were examined via 2% agarose gel electrophoresis, excised, and purified with the AxyPrep Gel Extraction Kit (Axygen Biosciences, San Diego, CA, USA). DNA concentration was measured using a Quantus™ Fluorometer (Promega, Madison, WI, USA). Equal-molar PCR products were pooled and sequenced (2 × 300 bp) on the Illumina MiSeq platform.

Sequencing data underwent standard processing. Demultiplexing and quality filtering were conducted using Trimmomatic, and read merging was performed by FLASH v1.2.7 [31]. Sequences were quality-trimmed using a sliding window approach, discarding reads with an average Phred score below 20 across any 50 bp segment. Reads shorter than 50 bp post-trimming were removed, and overlapping paired-end reads were combined if a minimum of 10 bp overlap was detected. Barcode matching permitted up to a pair of nucleotide discrepancies at the primer binding sites, and read orientation was corrected. OTU clustering at 97% similarity was conducted via UPARSE v7.1 [32], with chimera removal included. Taxonomic assignment was achieved using the RDP Classifier v2.2, aligned against the NT database [33]. Nontarget species (e.g., bacteria and viruses) were discarded. Detailed procedures for species filtering are provided in Figure S1.

### 2.4. Community-Level Data Acquisition and Analysis

#### 2.4.1. Fish Species Richness

Using presence–absence data, taxonomic-level species richness was assessed for both eDNA-based detection and the traditional survey method (TSM) [13]. Subsequently, for each sampling site, the proportion of species richness attributable to each method was computed by dividing the number of species identified by that method by the total number of unique species detected across both methods [14,29].

#### 2.4.2. Relative Abundance

Estimates of relative species abundances were based on OTU abundance matrices for eDNA data and on species count data obtained through the traditional survey method (TSM) [34,35]. In particular, for eDNA-based assessments, relative abundance at each site was determined by dividing the sequence count of each species by the total number of sequences obtained [34]. In contrast, for the TSM, relative abundance was calculated as the proportion of individuals of a given species relative to the total number of individuals recorded at the site [35].

#### 2.4.3. Dominant Species Analysis

Dominant species were identified using the Index of Relative Importance (IRI), as proposed by Pinkas et al. [36]. The IRI is calculated as follows:

IRI=(N+W) F

In the formula, N means the proportion of a given taxon among the captured specimens, W denotes its corresponding mass percentage, and F indicates the frequency of occurrence, defined as the proportion of samples in which the taxon appears relative to the total number of surveys conducted. Species were categorized based on their Index of Relative Importance (IRI) values as follows: those with IRI > 1000 were designated as dominant, values between 100 and 1000 indicated important species, IRI scores ranging from 10 to 100 were classified as common, those between 1 and 10 as occasional, and species with IRI < 1 were considered rare [36].

#### 2.4.4. Risk Screening Assessment

The Aquatic Species Invasiveness Screening Kit (AS-ISK) (available at www.cefas.co.uk/nns/tools/, accessed on 15 March 2025) assesses species invasiveness by generating a Basic Risk Assessment (BRA) score based on 49 biogeographic and biological questions, with six additional Climate Change Assessment (CCA) questions evaluating potential changes under future scenarios. The corresponding answers are detailed in Supplementary Material S2. The BRA score determines the risk level of introduction, spread, and impact of non-native species, while the BRA + CCA score can help assessors better understand the potential future development risks of non-native fish species [20]. ROC curve analysis, performed in SPSS 26.0, assessed AS-ISK’s accuracy by plotting sensitivity versus specificity, with the area under the curve (AUC) indicating classification accuracy [19]. Optimal thresholds were identified using Youden’s index, with a default value of 1 distinguishing low- and medium-risk species. Confidence factors (CF) were derived to evaluate assessment reliability, with CF values calculated based on assigned confidence levels [37]. A confidence factor (CF) was calculated based on the confidence levels (CL) assigned to individual responses within the risk screening framework (see Risk Screening). The CF was derived using the following formula:

$$CF=\Sigma (CL_{Q_{i}})/(4\times 55)(i=1,\ldots ,55)$$

where *CL_Qi_* denotes the confidence level assigned to question *Q_i_*, 4 represents the maximum possible score corresponding to a “very high” confidence rating, and 55 indicates the total number of assessment questions. Confidence factors were also computed independently for the BRA (Basic Risk Assessment) and the CCA (Climate Change Assessment) components [38].

### 2.5. Statistical Analyses

Statistical analyses were conducted using SPSS version 26.0 and Microsoft Excel 2019. Visualization of species composition, invasion severity, and confidence levels was carried out using Origin 2022, which was employed to generate pie charts, bar graphs, and line plots, respectively. Receiver Operating Characteristic (ROC) curves were constructed in GraphPad Prism 8, and statistical significance was evaluated based on the area under the curve (AUC), with *p* < 0.05 considered indicative of a statistically significant result.

## 3. Results

### 3.1. Community Profile in Xinglin Bay

**Figure 2:**
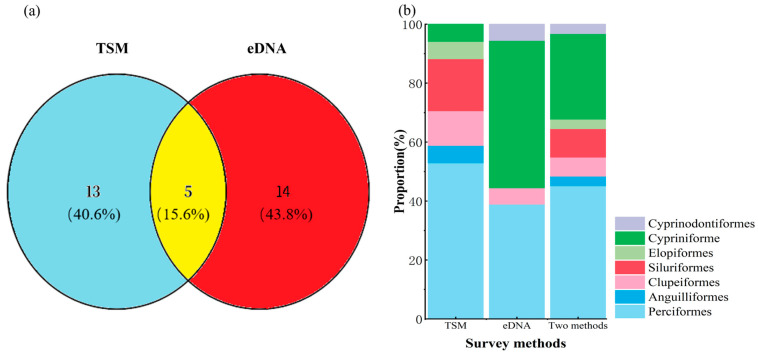
(**a**) Comparative species richness of fish identified via the traditional sampling method (TSM) and environmental DNA (eDNA) analysis. (**b**) Proportional representation of taxonomic order richness as detected independently by eDNA, TSM, and by the combination of both approaches.

A total of 18 fish species spanning seven orders and twelve families were recorded through traditional sampling methods (TSM). In contrast, environmental DNA (eDNA) yielded 335,541 sequence reads, leading to the identification of nineteen species across five orders and nine families. When data from both approaches were integrated, thirty-two distinct fish species belonging to eight orders and fifteen families were documented. Of these, five species (15.6%) were concurrently identified by both TSM and eDNA techniques (Table S1). Additionally, fourteen species (43.8%) were uniquely detected via eDNA, while thirteen species (40.6%) were exclusively recorded by TSM (Figure 2a; Table 1).

Within the TSM dataset, the most species-rich order was Perciformes, comprising nine species and representing 50% of the total. Siluriformes followed with three species (16.67%), while Clupeiformes contributed two species (11.11%). Each of the remaining orders—Cypriniformes, Mugiliformes, Elopiformes, and Anguilliformes—was represented by a single species, each constituting 5.56% (Figure 2b). In the eDNA dataset, Cypriniformes dominated with nine species (47.37%), followed by Perciformes with seven species (36.84%). Clupeiformes, Mugiliformes, and Cyprinodontiformes each contributed one species (5.26%). Upon merging the TSM and eDNA datasets, Perciformes emerged as the most diverse order with fourteen species (43.75%), followed by Cypriniformes with nine species (28.13%). Siluriformes included three species (9.38%), while Clupeiformes accounted for two (6.25%). Elopiformes, Anguilliformes, Mugiliformes, and Cyprinodontiformes were each represented by a single species (3.13%) (Figure 2b).

### 3.2. Relative Abundance

**Figure 3:**
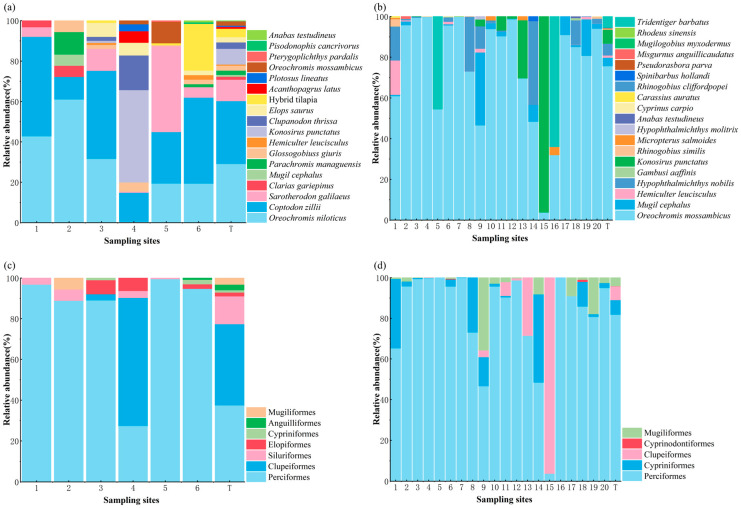
(**a**–**d**) Relative abundance (%) of fish taxa across sampling sites as determined by traditional sampling methods (TSM; panels **a**,**c**) and environmental DNA analysis (eDNA; panels **b**,**d**). Panels (**a**,**b**) represent species-level composition, while panels (**c**,**d**) show order-level composition. ‘T’ denotes the overall abundance at the reservoir-wide scale.

In terms of relative abundance, *Coptodon zillii* (Gervais, 1848) represented the most dominant species captured by traditional sampling methods (TSM), comprising 31.03% of the total catch, followed by *Oreochromis niloticus* (Linnaeus, 1758) at 35.13% (Figure 3a). The site-specific relative abundance of *C. zillii* using TSM was recorded as 49.91%, 11.11%, 43.56%, 14.29%, 25.46%, and 42.54% at Sites 1 through 6, respectively. Correspondingly, the relative abundance of *O. niloticus* at these sites was 42.86%, 61.11%, 31.86%, 0.57%, 19.44%, and 19.40% (Figure 3a).

Using the eDNA approach, *Oreochromis mossambicus* (Peters, 1852) exhibited the highest overall relative abundance, constituting 75.68% of all eDNA reads, followed by *Mugil cephalus* (Linnaeus, 1758) at 6.80% (Figure 3b). *O. mossambicus* predominated across nearly all sampling locations, with the exception of Sites 15 and 16, where its relative abundance dropped to 3.77% and 32.00%, respectively. The relative abundance of *O. mossambicus* varied significantly, ranging from a minimum of 3.77% (Site 15) to a peak of 99.88% (Site 4). At Site 16, *Tridentiger barbatus* (Günther, 1861) emerged as the dominant species with 64.00% relative abundance, whereas at Site 15, *Konosirus punctatus* (Temminck and Schlegel, 1846) was most prevalent, accounting for 96.23% (Figure 3b).

With respect to taxonomic composition by order, both methods consistently indicated Perciformes as the most abundant group, followed by Clupeiformes. Specifically, in TSM-derived data, Perciformes and Clupeiformes accounted for 82.76% and 10.97% of total individuals, respectively (Figure 3c). Similarly, eDNA-based estimates showed that Perciformes comprised 81.84%, while Cypriniformes represented 7.16% of the total reads (Figure 3d). At the family level, both methods consistently indicated Cichlidae as the most abundant group, accounting for 75.68% and 79.22% in the TSM and eDNA method, respectively (Figure S2).

### 3.3. Species Relative Importance Value

The fish specimens collected using traditional capture methods were systematically sorted and analyzed. The dominance of each species within the community was quantified using the Index of Relative Importance (IRI). The analysis revealed that the fish community in the Xinglin Bay was dominated by three species: *C. zillii* (IRI = 4834.50), *Sarotherodon galilaeus* (Linnaeus, 1758) (IRI = 4212.88), and *O. niloticus* (IRI = 3244.33), with *C. zillii* exhibiting the highest IRI value. In addition, six species were identified as important components of the community, ranked in descending order of IRI as follows: Hybrid tilapia (IRI = 436.45), *K. punctatus* (IRI = 415.92), *Clupanodon thrissa* (Linnaeus, 1758) (IRI = 309.33), *Elops saurus* (Linnaeus, 1766) (IRI = 241.65), *O. mossambicus* (IRI = 236.59), and *Glossogobius giuris* (Hamilton, 1822) (IRI = 236.22) (Table 1).

### 3.4. Body Length and Weight Characteristics of Fish

The body length and weight of each fish were analyzed based on the TSM. Only one fish species, *Clarias gariepinus* (Burchell, 1822), had an average body length exceeding 20 cm, with an average length of 25.70 cm and an average weight of 219.00 g. *E. saurus*, *K. punctatus*, and *C. thrissa* had average body lengths ranging from 15 to 20 cm, with respective average lengths of 18.16 cm, 17.40 cm, and 16.65 cm, and corresponding average weights of 42.28 g, 81.35 g, and 70.87 g. The average body lengths of *Hemiculter leucisculus* (Basilewsky, 1855), *Acanthopagrus latus* (Houttuyn, 1782), *S. galilaeus*, *O. niloticus*, *O. mossambicus*, *Parachromis managuensis* (Günther, 1867), hybrid tilapia, *Plotosus lineatus* (Thunberg, 1787), and *G. giuris* ranged from 10 to 15 cm. Their respective average lengths were 14.35 cm, 13.74 cm, 13.20 cm, 12.22 cm, 12.22 cm, 11.78 cm, 10.78 cm, 10.67 cm, and 10.29 cm, with corresponding average weights of 34.27 g, 89.61 g, 95.47 g, 77.12 g, 62.74 g, 73.70 g, 54.98 g, 10.95 g, and 22.61 g. *C. zillii* was the only species with an average body length less than 10 cm, with a mean length of 9.12 cm and a mean weight of 40.25 g. Only a single individual of *M. cephalus*, *Pterygoplichthys pardalis* (Castelnau, 1855), and *Anabas testudineus* (Bloch, 1792) was found, with body lengths of 43.50 cm, 18.60 cm, and 12.70 cm, and corresponding weights of 1647.00 g, 121.80 g, and 74.50 g, respectively. One individual of *Pisodonophis cancrivorus* (Richardson, 1848) was also observed, with an anal length of 19.50 cm and a weight of 153.80 g, as shown in Table 2.

### 3.5. Assessment of Invasion Risk for Non-Native Fish

**Figure 4:**
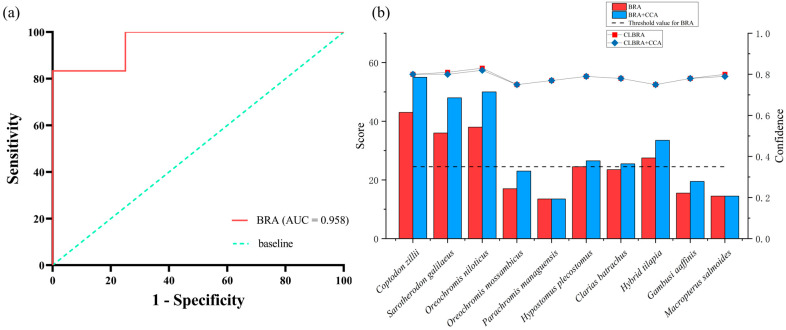
(**a**) Receiver-operating-characteristic (ROC) analysis illustrating the diagnostic performance of the AS-ISK evaluation for ten non-native fish species recorded in Xinglin Bay; (**b**) panel b displays the AS-ISK risk scores as bar charts with overlaid confidence levels shown as a line graph; the horizontal reference line represents the threshold identified by Youden’s J statistic.

Receiver Operating Characteristic (ROC) curve analysis for the BRA score yielded an Area Under the Curve (AUC) of 0.958, indicating excellent discriminatory ability (Figure 4a). Based on Youden’s J statistic, a threshold value of 24.5 was determined and subsequently employed to calibrate the BRA-based risk classification. Following calibration, BRA scores were categorized into three risk intervals: low risk (−20 to 1), medium risk (1 to 24.5), and high risk (24.5 to 68). According to this classification scheme, half of the evaluated fish species (five out of ten) were assigned to the high-risk category, while the remaining five species were designated as medium risk (Table 3; Figure 4b). Among those scoring above the high-risk threshold (≥24.5), the species ranked from highest to lowest BRA scores were *C. zillii*, *O. niloticus*, *S. galilaeus*, Hybrid tilapia, and *P. pardalis* (Table 3).

Across the 55 assessment criteria, the mean confidence factor for the BRA scores was 0.78 ± 0.03, while that for the combined BRA + CCA assessment was 0.78 ± 0.02. In both cases, the average confidence factor exceeded 0.75, suggesting a moderate to high level of assessor confidence in the overall evaluation results.

## 4. Discussion

### 4.1. Application of eDNA for Fish Community Assessment in Xinglin Bay

This study combined TSM with eDNA to detect a total of 32 fish species. Specifically, TSM identified 18 species, whereas eDNA detected 19 species. Compared to TSM, eDNA detected 14 additional species, including two non-native species, *G. affinis* and *M. salmoides*, with relatively low abundances of 0.16% and 1.03%, respectively. Balasingham et al. (2018) also highlighted the high sensitivity and strong detection capability of eDNA in fish monitoring, particularly for species with low abundance and high crypticity [39]. However, in this study, thirteen fish species detected by TSM were not identified through eDNA, including eleven mid- to bottom-dwelling species (e.g., *G. giuris*, *P. cancrivorus*, *C. zillii*, *P. lineatus*, *O. niloticus* etc.) and two pelagic species. The failure of eDNA to detect these species may be related to the sampling strategy. The average depth in Xinglin Bay was approximately 2.5 m, with only surface water samples collected for eDNA analysis. This likely failed to capture DNA released by mid- to bottom-dwelling fish, resulting in species inhabiting these layers accounting for as much as 84.61% of the species undetected by eDNA. Jeunen et al. (2020) used eDNA to sample three vertical strata at depths of 0 m, 4 m, and 15 m along steep rock walls in New Zealand fiords, demonstrating that stratified sampling more comprehensively captures the vertical spatial distribution of fish communities and recommending mixed or multilayer water sampling strategies in future studies to improve detection coverage [40]. Moreover, for the two undetected pelagic fish, their abundances were both below 4%. Although eDNA possesses high sensitivity for detecting low-abundance species, it does not guarantee detection of all such species. Detection results may still be influenced by multiple factors including DNA concentration, degradation rate, and amplification efficiency [41]. On the other hand, the sensitivity of eDNA methods is also affected by the specificity and sensitivity of the primers used. Employing a single primer pair may fail to cover all species, resulting in partial taxonomic omissions [42]. Therefore, the introduction of multiple primer pairs is recommended in future fish community studies to enhance taxonomic resolution. Regarding fish spatial distribution, the results found that fish communities assessed by both methods reflected differences in habitat characteristics. The relative abundance of fish inhabiting seawater and brackish water near the high-salinity area in the southern estuarine region (Site 4 for TSM; Sites 15 and 16 for eDNA) was notably higher than at other sites [24]. In summary, eDNA showed considerable potential for application in brackish ecosystems, enabling higher species detection rates and differentiation of heterogeneity in fish communities across different habitats. In the future, integrating multi-layer sampling with the use of multiple primer sets is expected to further enhance the accuracy and reliability of eDNA in fish community assessments.

### 4.2. Fish Community Structure and Risk Assessment of Non-Native Fish Species in Xinglin Bay

A total of 32 fish species were detected using a combination of TSM and eDNA, among which ten were non-native. The relative abundance of tilapia reached 80.75% and 75.68% using the TSM and eDNA methods, respectively. Furthermore, IRI results showed that among the ten non-native fish species, *C. zillii*, *S. galilaeus*, and *O. niloticus* were dominant species, and the hybrid tilapia and *O. mossambicus* were important species. Wang et al. (2024) assessed the fish community structure in Xinglin Bay in January 2023 and detected 12 species, with Perciformes accounting for 33.32%, and three non-native fish species, where tilapia accounted for 56.91% and 89.80% in the TSM and eDNA methods, respectively [24]. Our findings and previous studies indicated that the community composition was similar and that tilapia invasion was severe. In addition, the species richness showed a significant increase. One possible reason is the higher water temperature in summer, which may have promoted population growth and activity range expansion, increasing species detectability [43,44]. Another reason is that this study included more sampling sites and covered more microhabitat types than previous research, improving the comprehensiveness of species detection [45]. Therefore, this study indicated that non-native species have become dominant in Xinglin Bay, with particular attention needed for the highly abundant tilapia.

In addition, the AS-ISK assessment demonstrated that all non-native fish species are classified within medium- and high-risk categories, with five identified as high-risk species—four of which belong to tilapia. Tilapia has been widely and successfully introduced globally due to its broad environmental tolerance, rapid growth, and high reproductive capacity, traits that also confer it a high invasive potential [46]. Additionally, tilapias are generalist feeders that consume large quantities of herbivorous invertebrates, fish eggs, and juveniles, exerting intense competition on native resources and impacting the growth and reproduction of other biotic communities, thereby altering interspecific interactions and the structure of the food web [47]. Moreover, the excretion and nest-building behavior of tilapias can increase eutrophication levels and degrade water quality [48]. For instance, the invasion of *O. niloticus* in the Murray–Darling Basin of Australia has led to a sharp decline in native fish populations and disrupted nutrient cycling in the aquatic environment [49]. Martin et al. (2010) demonstrated through laboratory experiments that introduced *O. niloticus* directly displaced functionally similar native fish (e.g., *Lepomis miniatus*) and altered their habitat use preferences, thereby affecting native species’ survival and foraging behavior [50]. Shuai et al. (2023) reported the impacts of *O. niloticus* invasion on dietary competition and the trophic positions of native piscivores in the lower Pearl River of China, in which the native piscivores shift to feeding on zooplankton or aquatic insects, resulting in shortened food chains and compressed niche diversity [51]. In addition, numerous urban and coastal freshwater systems across southern China have been heavily impacted by tilapia invasions [52]. Notably, the inland urban freshwater bodies of Guangdong Province, including the Pearl River Basin, have experienced extensive colonization by *C. zillii* and *O. niloticus*, which have significantly disrupted native fish communities by impairing their growth, reproduction, and ecological dynamics [53]. Therefore, based on the dominant species analysis and AS-ISK assessment, this study highlights the high abundance and significant invasion threat of tilapia, recommending it as a priority target for invasive species control in Xinglin Bay to curb its spread and protect native biodiversity. Given the fast reproduction and strong tolerance of tilapia, long-term monitoring of fish communities in Xinglin Bay is also advised to support effective management strategies of non-native fish.

### 4.3. Recommendations for Controlling and Managing Tilapia in Xinglin Bay

Tilapia (including *C. zillii*, *O. niloticus*, and *S. galilaeus*), renowned for their strong ecological adaptability, have become highly invasive in coastal cities across southern China, including Fujian, Guangdong, and Hainan, posing serious threats to the health of local aquatic ecosystems [54]. The effective prevention and management of tilapia have become critical tasks for the sustainable development of aquatic ecosystems in coastal cities of southern China. To date, control strategies for tilapia have primarily included chemical, biological, and physical approaches [55]. Among chemical control methods, piscicides can rapidly eliminate tilapia populations. For example, Nico et al. (2015) reported that the application of rotenone/CFT Legumine can rapidly eliminate over 98% of invasive tilapia; however, the associated chemical toxicity poses substantial risks to non-target organisms in the food web and can severely degrade water quality [56]. In addition, biological methods for controlling tilapia are currently primarily based on the introduction and stocking of predatory fish [57]. However, we believe that this method may be unsuitable for effective tilapia control in Xinglin Bay. On the one hand, the composition of the fish community indicated an absence of native piscivorous species with the capacity to prey on invasive tilapia in this aquatic system. Introducing species that do not exist in the original aquatic environment may result in their failure to survive due to poor environmental adaptability. This not only leads to ineffective tilapia control but may also cause a significant waste of human and material resources. On the other hand, biological control is typically a long-term ecological restoration process. Given the annual occurrences of mass fish mortality and the persistently poor water quality conditions in Xinglin Bay [23], the introduction of additional fish species may exacerbate fish mortality, potentially resulting in counterproductive outcomes. Therefore, we consider that conducting regular and targeted removal operations, especially during or prior to the breeding season, may represent a practical and effective strategy for controlling tilapia populations at this stage in Xinglin Bay. DJ et al. (2012) have effectively mitigated tilapia by implementing a combination of fishing gears including gill nets, fyke nets, electrofishing, and seine nets in Australia [58]. Therefore, it is recommended to implement targeted harvesting of tilapia in regions with higher abundance of tilapia (specifically non-estuarine regions), particularly during or immediately preceding the breeding season, as a primary strategy for the effective control and management of tilapia.

## 5. Conclusions

By integrating TSM with eDNA, this study systematically assessed the fish community structure and invasion status of non-native species in Xinglin Bay, Xiamen, and evaluated their invasion risks using the AS-ISK tool. A total of thirty-two fish species were detected, among which ten species were non-native. The relative abundance of these non-native fish species accounted for 80.75% using TSM and 75.68% using eDNA. Compared to TSM, eDNA revealed a greater number of species, particularly those with low abundance and high crypticity. The AS-ISK results showed that five of the ten non-native fish species were classified as high-risk (including four species of tilapia), while the remaining were assessed as medium-risk species. Thus, population reduction through targeted harvesting of tilapia is recommended as the primary control strategy. Furthermore, this study demonstrated the effectiveness of eDNA in monitoring fish communities and non-native fish in brackish ecosystems, offering methodological insights for monitoring of non-native species in lentic brackish ecosystems.

## Figures and Tables

**Table 1:** The IRI values of fish in Xinglin Bay.

Species	Number	Weight	N	W	IRI
*Coptodon zillii*	214	8614.33	30.31%	18.03%	4834.50
*Sarotherodon galilaeus*	139	10,719.52	19.69%	22.44%	4212.88
*Oreochromis niloticus*	114	10,883.95	16.15%	22.78%	3244.33
Hybrid tilapia	34	1869.26	4.82%	3.91%	436.45
*Konosirus punctatus*	80	6507.9	11.33%	13.62%	415.92
*Clupanodon thrissa*	32	2267.72	4.53%	4.75%	309.33
*Elops saurus*	21	887.78	2.97%	1.86%	241.65
*Oreochromis mossambicus*	26	1631.33	3.68%	3.42%	236.59
*Glossogobius giuris*	15	339.17	2.12%	0.71%	236.22
*Clarias gariepinus*	3	657	0.42%	1.38%	60.01
*Mugil cephalus*	1	1647	0.14%	3.45%	59.83
*Acanthopagrus latus*	10	896.1	1.42%	1.88%	54.87
*Parachromis managuensis*	4	294.8	0.57%	0.62%	39.46
*Hemiculter leucisculus*	4	137.09	0.57%	0.29%	28.45
*Plotosus lineatus*	6	65.7	0.85%	0.14%	16.46
*Pisodonophis cancrivorus*	1	153.8	0.14%	0.32%	7.73
*Pterygoplichthys pardalis*	1	121.8	0.14%	0.25%	6.61
*Anabas testudineus*	1	74.5	0.14%	0.16%	4.96

Note: N represents numerical percentage; W denotes weight percentage; IRI stands for the Index of Relative Importance.

**Table 2:** Body length and body weight of fish in Xinglin Bay.

Species	Body Length/Anal Length (cm)		Body Weight (g)	
	Mean ± S.D.	Range	Mean ± S.D.	Range
*Clarias gariepinus*	25.70 ± 4.48	21.50–31.90	219.00 ± 101.64	105.30–352.00
*Elops saurus*	18.16 ± 2.40	13.40–22.50	42.28 ± 15.87	18.30–81.10
*Konosirus punctatus*	17.40 ± 3.09	9.60–24.20	81.35 ± 36.74	13.00–196.70
*Clupanodon thrissa*	16.65 ± 3.96	8.80–23.60	70.87 ± 43.51	10.40–174.90
*Hemiculter leucisculus*	14.35 ± 1.91	12.40–17.50	34.27 ± 4.94	27.19–40.98
*Acanthopagrus latus*	13.74 ± 1.06	11.90–15.30	89.61 ± 23.04	52.40–126.10
*Sarotherodon galilaeus*	13.20 ± 3.08	3.30–17.00	95.47 ± 46.01	4.00–171.20
*Oreochromis niloticus*	12.22 ± 4.77	3.10–23.20	77.12 ± 58.40	1.40–232.74
*Oreochromis mossambicus*	12.22 ± 1.31	9.40–15.40	62.74 ± 26.02	28.84–152.93
*Parachromis managuensis*	11.78 ± 3.34	9.40–17.50	73.70 ± 64.35	35.30–185.10
*Hybrid tilapia*	10.78 ± 2.71	8.50–24.50	54.98 ± 51.71	26.64–327.50
*Plotosus lineatus*	10.67 ± 1.09	9.00–12.00	10.95 ± 3.20	6.30–15.10
*Glossogobiuss giuris*	10.29 ± 2.18	5.50–14.70	22.61 ± 11.99	3.70–52.60
*Coptodon zillii*	9.12 ± 4.39	2.10–17.50	40.25 ± 35.11	0.50–137.70
*Mugil cephalus*	-	43.50	-	1647.00
*Pterygoplichthys pardalis*	-	18.60	-	121.80
*Anabas testudineus*	-	12.70	-	74.50
*Pisodonophis cancrivorus*	-	19.50	-	153.80

Note: “-” indicates that only one individual was sampled and measured. The anal length of *Pisodonophis cancrivorus* was measured in this study.

**Table 3:** Assessment of Non-Native Fish in Xinglin Bay Using the Aquatic Species Invasiveness Screening Kit (AS-ISK).

Species	A Priori Categorization	Results			Confidence		
		BRA	Level	BRA + CCA	BRA + CCA	BRA	CCA
*Coptodon zillii*	Y	43	High	55	0.78	0.78	0.75
*Sarotherodon galilaeus*	Y	35	High	48	0.79	0.79	0.79
*Oreochromis niloticus*	Y	38	High	50	0.80	0.81	0.75
Hybrid tilapia	Y	27.5	High	33.5	0.77	0.77	0.75
*Oreochromis mossambicus*	N	17	Medium	23	0.75	0.75	0.75
*Parachromis managuensis*	Y	13.5	Medium	13.5	0.82	0.83	0.75
*Pterygoplichthys pardalis*	Y	24.5	High	26.5	0.80	0.80	0.75
*Clarias gariepinus*	Y	23.5	Medium	25.5	0.75	0.75	0.75
*Gambusia affinis*	N	15.5	Medium	19.5	0.78	0.78	0.75
*Micropterus salmoides*	N	14.5	Medium	14.5	0.80	0.79	0.75

Note: A priori invasion status is indicated as N (non-invasive) or Y (invasive). Assessment outcomes include results from the Basic Risk Assessment (BRA), the combined BRA and Climate Change Assessment (BRA + CCA), and the Δ (Delta) score, defined as the difference between the BRA + CCA and BRA scores. Confidence level (CL) represents the average confidence in the responses. Final risk categorization is based on calibrated score intervals: Low risk (score < 1), Medium risk (1 ≤ score < 24.5), and High risk (24.5 ≤ score < 68).

## Data Availability

The Raw sequence reads detected by eDNA are deposited in the NCBI Sequence Read Archive database BioProject ID: PRJNA1276155 (https://www.ncbi.nlm.nih.gov/sra/PRJNA1276155, accessed on 15 March 2025). Part of the data presented in this study are available in the Supplementary Material. The remaining data presented in this study are available upon reasonable request from the corresponding author.

## Supplementary Materials

The following supporting information can be downloaded at: https://www.mdpi.com/article/10.3390/biology14080988/s1, File S1: Table S1. species detected in the Xinglin Bay using eDNA and TSM. Figure. S1 Species-filtering steps identifying target species. Figure S2. Relative abundance (%) of fish at the family level with TSM (a) or eDNA (b) in per sampling site. File S2: Screening 1–10.
