# Supplementary material for: Assessment of Fish Community Structure and Invasion Risk in Xinglin Bay, China
Source: Biology (Basel). 2025 Aug 4;14(8):988. doi: 10.3390/biology14080988 (PMC12384033; doi:10.3390/biology14080988)
Supplement: Supplementary file 1 [file biology-14-00988-s001.zip › Supplementary material 1(1).pdf]

Article

Assessment of Fish Community Structure and Invasion Risk in Xinglin Bay, China

Shilong Feng[1,2#], Xu Wang[1,2#], Liangmin Huang[1,2], Jiaqiao Wang[1,2], Lin Lin[1,2], Jun Li[1,2], Guangjie Dai[3], Qianwen Cai[3], Haoqi Xu[1,2], Yapeng Hui[1,2], and Fenfen Ji[1,2]*

1 Fisheries College, Jimei University, Xiamen 361021, China

2 FuJian Provincial Key Laboratory of Marine Fishery Resources and Eco-Environment, Xiamen 361021, China

3 Agriculture, Rural and Water Resources Bureau of Jimei District, Xiamen 361022, China

# These authors contributed equally to this work

*Address correspondence to Fenfen Ji, Ph.D.
Fisheries College, Jimei University, 43 Yindou Road, Jimei District, Xiamen 36 1021, Chin
Tel.: +86-0592-6181054
Fax: +86-0592-6181476
Email: (fenfenji@jmu.edu.cn)

Table S1. species detected in the Xinglin Bay using eDNA and TSM

**species**	**Gunes**	**Family**	**Order**	**TSM**	**eDNA**
Sarotherodon galilaeus	Oreochromis	Cichlidae	Perciformes	+	-
Parachromis managuensis	Parachromis	Cichlidae	Perciformes	+	-
Clarias gariepinus	Clarias	Clariidae	Siluriformes	+	-
Konosirus punctatus	Konosirus	Clupeidae	Clupeiformes	+	+
Hemiculter leucisculus	Hemiculter	Cyprinidae	Cypriniformes	+	+
Elops saurus	Elops	Elopidae	Elopiformes	+	-
Clupanodon thrissa	Clupanodon	Clupeidae	Clupeiformes	+	-
Acanthopagrus latus	Acanthopagrus	Sparidae	Perciformes	+	-
Pisodonophis cancrivorus	Pisodonophis	Ophichthidae	Anguilliformes	+	-
Plotosus lineatus	Plotosus	Plotosidae	Siluriformes	+	-
Oreochromis niloticus	Oreochromis	Cichlidae	Perciformes	+	-
Coptodon zillii	Oreochromis	Cichlidae	Perciformes	+	-
Glossogobiuss giuris	Glossogobius	Gobiidae	Perciformes	+	-
Hybrid tilapia	Oreochromis	Cichlidae	Perciformes	+	-
Mugil cephalus	Mugil	Mugilidae	Mugiliformes	+	+
Pterygoplichthys pardalis	Pterygoplichthys	Loricariidae	Siluriformes	+	-
Anabas testudineus	Anabas	Anabantidae	Perciformes	+	+
Oreochromis mossambicus	Oreochromis	Cichlidae	Perciformes	+	+
Hypophthalmichthys nobilis	Hypophthalmichthys	Cyprinidae	Cypriniformes	-	+
Carassius auratus	Carassius	Cyprinidae	Cypriniformes	-	+
Rhinogobius similis	Rhinogobius	Gobiidae	Perciformes	-	+
Cyprinus carpio	Cyprinus	Cyprinidae	Cypriniformes	-	+
Micropterus salmoides	Micropterus	Centrarchidae	Perciformes	-	+
Gambusi aaffinis	Gambusia	Poeciliidae	Cyprinodontiformes	-	+
Hypophthalmichthys molitrix	Hypophthalmichthys	Cyprinidae	Cypriniformes	-	+

Pseudorasbora parva	Pseudorasbora	Cyprinidae	Cypriniformes	-	+
Misgurnus anguillicaudatus	Misgurnus	Cobitidae	Cypriniformes	-	+
Mugilogobius myxodermus	Mugilogobius	Gobiidae	Cypriniformes	-	+
Rhodeus sinensis	Rhodeus	Cyprinidae	Cypriniformes	-	+
Rhinogobius cliffordpopei	Rhinogobius	Gobiidae	Perciformes	-	+
Spinibarbus hollandi	Spinibarbus	Cyprinidae	Cypriniformes	-	+
Tridentiger barbatus	Tridentiger	Gobiidae	Perciformes	-	+

Note: TSM and eDNA represent the traditional survey method and eDNA metabarcoding, respectively. "-" indicates species absent fromTSM and eDNA; "+" indicates species present from TSM and eDNA

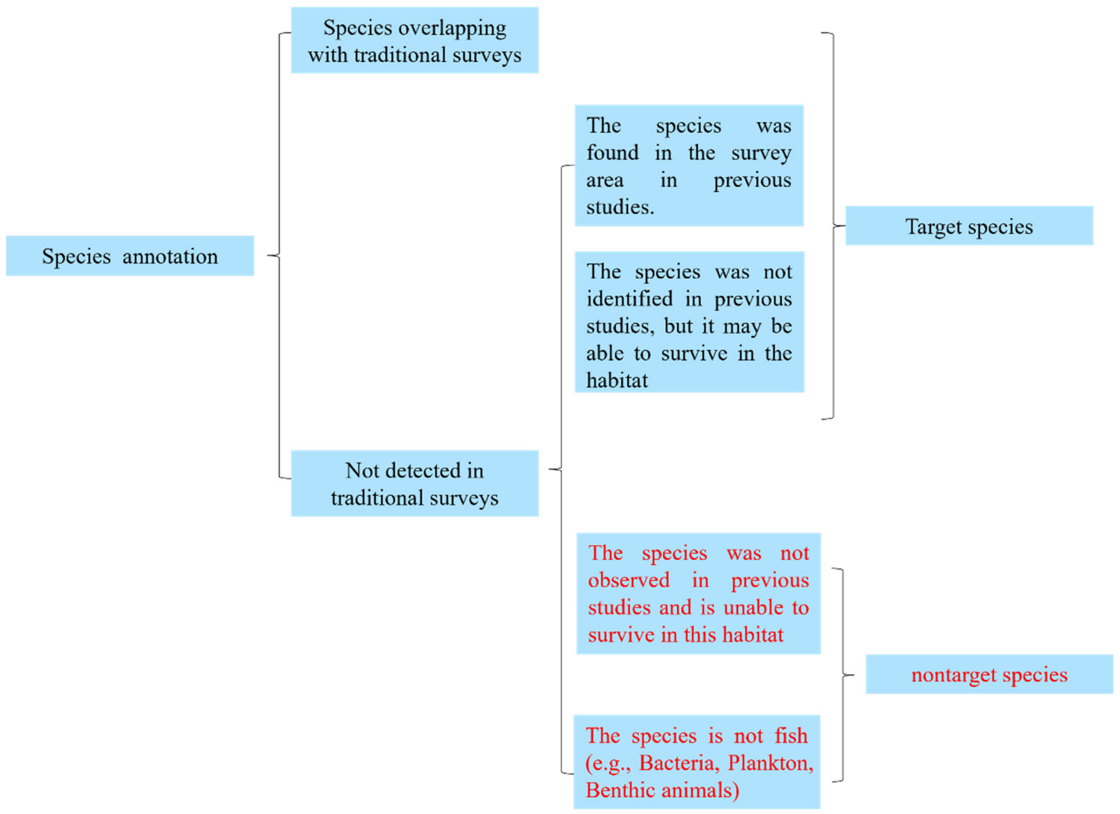

Figure. S1 Species-filtering steps identifying target species

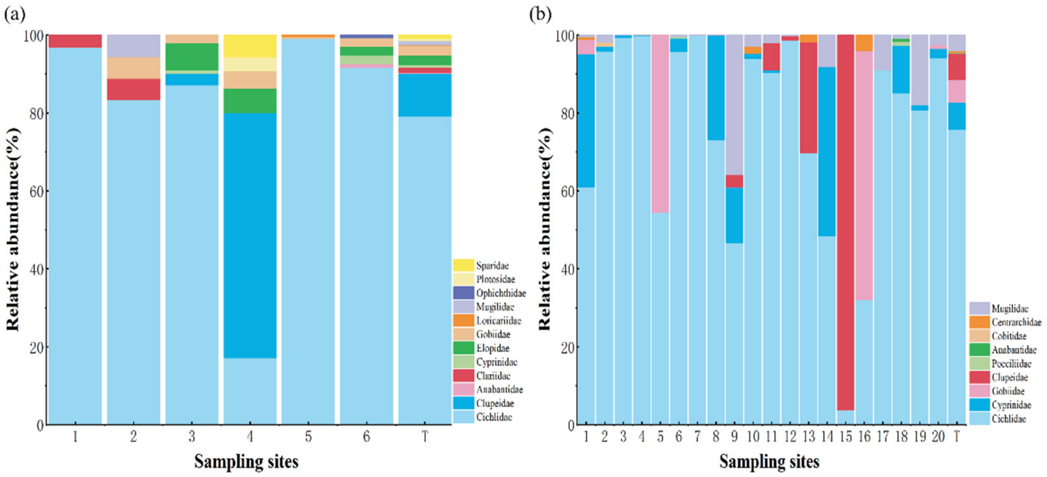

Figure S2. Relative abundance (%) of fish at the family level with TSM (a) or eDNA (b) in per sampling site
